# Reduced responsiveness is an essential feature of chronic fatigue syndrome: A fMRI study

**DOI:** 10.1186/1471-2377-6-9

**Published:** 2006-02-22

**Authors:** Masaaki Tanaka, Norihiro Sadato, Tomohisa Okada, Kei Mizuno, Tetsuya Sasabe, Hiroki C Tanabe, Daisuke N Saito, Hirotaka Onoe, Hirohiko Kuratsune, Yasuyoshi Watanabe

**Affiliations:** 1 Department of Physiology, Osaka City University Graduate School of Medicine, 1-4-3 Asahimachi, Abeno-ku, Osaka 545-8585, Japan; 2 Division of Cerebral Integration, Department of Cerebral Research, National Institute for Physiological Sciences, 38 Nishigonaka, Myodaiji, Okazaki, Aichi 444-8585, Japan; 3 Japan Science and Technology Corporation (JST)/Research Institute of Science and Technology for Society (RISTEX), 4-1-8 Honcho, Kawaguchi, Saitama 332-0012, Japan; 4 Department of Oral Physiology, Osaka University Graduate School of Dentistry, 1-8 Yamadaoka, Suita, Osaka 565-0871, Japan; 5 Department of Psychology, Tokyo Metropolitan Institute for Neuroscience, 2-6 Musashidai, Fuchu, Tokyo 183-8526, Japan; 6 Department of Health Science, Faculty of Health Science for Welfare, Kansai University of Welfare Sciences, 3-11-1 Asahigaoka, Kashihara, Osaka 582-0026, Japan

## Abstract

**Background:**

Although the neural mechanism of chronic fatigue syndrome has been investigated by a number of researchers, it remains poorly understood.

**Methods:**

Using functional magnetic resonance imaging, we studied brain responsiveness in 6 male chronic fatigue syndrome patients and in 7 age-matched male healthy volunteers. Responsiveness of auditory cortices to transient, short-lived, noise reduction was measured while subjects performed a fatigue-inducing continual visual search task.

**Results:**

Responsiveness of the task-dependent brain regions was decreased after the fatigue-inducing task in the normal and chronic fatigue syndrome subjects and the decrement of the responsiveness was equivalent between the 2 groups. In contrast, during the fatigue-inducing period, although responsiveness of auditory cortices remained constant in the normal subjects, it was attenuated in the chronic fatigue syndrome patients. In addition, the rate of this attenuation was positively correlated with the subjective sensation of fatigue as measured using a fatigue visual analogue scale, immediately before the magnetic resonance imaging session.

**Conclusion:**

Chronic fatigue syndrome may be characterised by attenuation of the responsiveness to stimuli not directly related to the fatigue-inducing task.

## Background

Chronic fatigue syndrome (CFS) is a disorder characterised by profound disabling fatigue that persists for at least 6 months without relief and is not lessened by ordinary rest [1]. Patients with CFS have substantial impairment of functional status, resulting in significant personal and economic morbidity [2,3]. Several symptoms reported by CFS patients, including impaired concentration, attention, and memory abilities [4,5], suggest that central nervous system (CNS) may be involved in the pathophysiology of CFS.

Magnetic resonance imaging (MRI) studies have shown anatomical anomalies of cortical [6,7] and subcortical [8-10] brain regions of CFS patients. Other studies using single-photon emission computed tomography have demonstrated reduced regional cerebral blood flow throughout the brain of CFS patients [11,12]. A positron emission tomography study confirmed reduced level of cerebral blood flow [13], and CFS patients also showed reduced cerebral glucose metabolism in some brain regions [14,15]. Therefore, overall, neuroimaging studies are generally consistent in demonstrating abnormalities of CNS in CFS patients.

Functional magnetic resonance imaging (fMRI) studies have also shown abnormal neuroimaging data in CFS patients. During motor imagery task, CFS patients evoked stronger responses in visually related structures relative to normal subjects [16]. When complex auditory information processing was required, CFS patients utilised more extensive regions of the network associated with verbal working memory system than normal subjects [17]. Thus, pattern of BOLD signal change of CFS subjects may be a reflection of the networks operation under conditions of increased mental effort [18-21] in order to overcome the experience of mental fatigue [17].

These previous studies only focused on static change under the condition without fatigue-load. Responsiveness can be defined as BOLD signal change responded by some stimuli in each brain region as measured using fMRI, and amplitudes of motor evoked potential elicited by transcranial magnetic stimulation were transiently decreased after exercise, indicating fatigue of motor pathways in the CNS [22]. Accordingly, CNS fatigue may induce reduced responsiveness. We hypothesised that, since CFS patients show marked fatigability in their daily activities, reduced responsiveness would be seen during fatigue-inducing period. Therefore, for further clarification of the neural mechanism of CFS, it may be essential to investigate brain responsiveness of CFS patients during a fatigue-load.

fMRI studies on human normal volunteers have demonstrated that continual visual stimuli from flashing goggles causes a decrease in signal intensity in the visual cortex [23-25], and that odorant-induced activity in the olfactory cortex decreases following prolonged odorant stimuli [26,27]. There is a problem to utilise these tasks as fatigue-inducing ones, since, in these studies, it is not clear whether subjects felt fatigue sensation during continual stimulation tasks. Therefore, we selected a task by which subjects could have fatigue sensation: A prolonged continual visual search task. This task was modified one of Advanced Trail Making Test, which can assess the degree of fatigue [28]. Many workers feel fatigue sensation by performing long-time personal computer works. Accordingly, fatigue-inducing visual search task was simulated to fatigue-inducing daily office works.

Fatigue can be induced without task-specific manner. In addition, responsiveness of task-dependent brain regions may be modulated by some confounds (e.g., mental efforts, attention, or learning). Thus, in order to clarify the neural mechanism of CFS using fMRI, it may be insufficient to evaluate the responsiveness of only task-dependent brain regions; since responsiveness of task-independent brain regions is not influenced by those confounds, it may also be necessary to evaluate the responsiveness of task-independent brain regions. However, this was difficult to achieve using fMRI. Recently, we established a new method to obtain the responsiveness of auditory cortices by transient reduction of the fMRI acquisition noise [29]. This can be used to monitor the responsiveness of the auditory cortices simultaneously to activation by other non-auditory tasks, facilitating evaluation of a cross-modal interaction. Therefore, by using this method, we may be able to evaluate the responsiveness of both task-independent and task-dependent brain regions while subjects conduct a fatigue-inducing task. We evaluated the responsiveness of task-independent and -dependent brain regions simultaneously during a fatigue-inducing visual search task in normal and CFS patients groups using a 3.0 tesla MR scanner.

## Methods

### Subjects

The patient group consisted of 7 men with CFS (age, 30.4 ± 4.8 years; mean ± SD). All values are expressed as mean ± SD, unless described elsewhere. They were outpatients of the Department of Hematology and Oncology of Osaka University. A diagnosis of CFS was made based on clinical criteria proposed previously [1]. None of these patients were able to carry out normal occupational, educational, social, or personal activities more than 2 days a week because of severe fatigue sensation. Duration of CFS was 24.2 ± 15.7 months. CFS patients who manifested psychiatric comorbidity (e.g. depression) were excluded from the study. Seven age-matched healthy male volunteers (26.1 ± 6.3 years of age) were recruited as a control. There was no history of psychiatric or neurological illness among the normal subjects. None of the normal or CFS subjects were taking medication known to affect cerebral blood flow, all had normal auditory function, and they were all right-handed according to the Edinburgh handedness inventory [30]. We excluded data of 1 CFS patient from the analyses on the basis of difficulties related to task performance. The research protocol was approved by the ethics committee of the National Institute for Physiological Sciences, and all subjects gave their written informed consent for the study.

### Task

**Figure 1:**
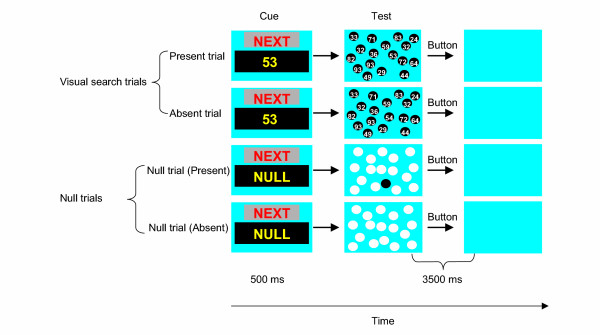
Time course of the stimulus display sequence of the task trials. For present and absent trials, a randomly selected digit from 20 to 99 was presented on the centre of the viewing screen for 500 ms, followed by a test sequence of digits (targets) presented for 3,500 ms. In a given trial, subjects judged whether the digit first presented was among the targets. If they believed the presented target digit matched the first digit, subjects pressed the left button (present trial); if not, they pressed the right button (absent trial). There was no time interval between trials. For the null trial, the word "NULL" was presented on the centre of the viewing screen for 500 ms, followed by a test sequence presented for 3,500 ms. Subjects judged whether there was a black circle among the white circles. When a black circle was present among the white ones, subjects pressed the left button; and when all of the circles were white, they pressed the right button. There was no time interval between the trials.

**Figure 2:**
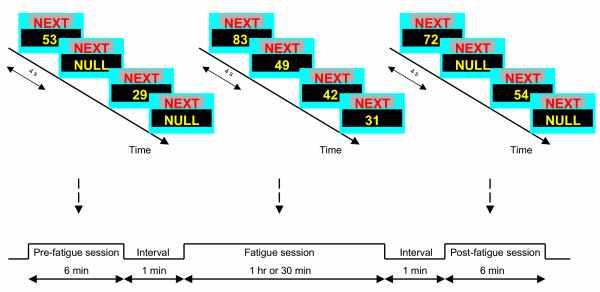
Experimental procedure. Experiment included 3 sessions: Pre-fatigue, fatigue, and post-fatigue sessions. Subjects performed pre-fatigue, fatigue, and post-fatigue sessions lying on the MRI scanner table with both ears plugged. The time interval between 2 successive sessions was 1 min. For the fatigue session (fatigue-inducing period), normal subjects performed visual search trials (present or absent trials) for 1 hour, while CFS patients performed visual search trials for 30 min. During this period, continual visual search trials were performed; null trials were not included. In the pre- or post-fatigue sessions, subjects performed visual search or null trials for 6 min.

Before the fMRI experiments, all subjects completed visual search tasks 3 times, for 6 min each, to evaluate their capacity to perform this task. The number of targets used in the visual search tasks in the subsequent fMRI experiments was determined separately for each individual based on their performance in the pre-imaging visual search tasks, thus minimizing possible confounding effects of a difference in task difficulty among the subjects. We determined the number of targets for which subjects could provide correct answers to visual search trials in approximately 90% of cases. The number of targets was 22.6 ± 2.0 for the normal subjects, whereas that was 19.0 ± 1.3 for the CFS patients. During experiments, subjects performed "present", "absent", or "null" trials (Figure 1), and the experiments consisted of 3 sessions: pre-fatigue, fatigue, and post-fatigue sessions (Figure 2). Each session included 2 or 3 experimental conditions: "present" and "absent" trials or "present", "absent", and "null" trials. For present and absent trials, a randomly selected digit from 20 to 99 was presented on the centre of the viewing screen for 500 ms, followed by a test sequence of digits (targets) presented for 3,500 ms. In a given trial, subjects judged whether the digit first presented was among the targets. If they thought the presented target digit matched the first digit, subjects should press the left button (present trial); if not, they should press the right button (absent trial). There was no time interval between trials. For the null trial, the word "NULL" was presented on the centre of the viewing screen for 500 ms, followed by a test sequence presented for 3,500 ms. Subjects judged whether there was a black circle among the white circles. When a black circle was presented among the white ones, subjects should press the left button; and when all of the circles were white, they should press the right button. Subjects performed pre-fatigue, fatigue, and post-fatigue sessions lying on the MRI scanner table with both ears plugged. The time interval between 2 successive sessions was approximately 1 min. We determined session time of fatigue-inducing period from the preliminary studies. For the fatigue session (fatigue-inducing period), normal subjects performed visual search trials (present or absent trials) for 1 hour. After considering the physical and mental condition of the CFS patients, we determined that 30 min was a suitable period for these patients to perform the trials. During the fatigue-inducing period, present or absent trials were given randomly and the occurrence of each trial was equal. Also during this period, only continual visual search trials were performed; null trials were not included as they might have enabled subjects to recover from fatigue to some extent. During the pre- and post-fatigue sessions, present, absent, and null trials were presented randomly, and the occurrence of each trial was equal. In the pre- or post-fatigue sessions, subjects performed trials for 6 min. During scanning, stimuli were generated by a personal computer and projected onto a semitransparent screen from a liquid crystal display projector (DLA-M200L, Victor, Yokohama, Japan). The subjects saw the stimuli through a tilted mirror attached to the head coil of the scanner. The visual angle of each target used as a stimulus was approximately 1°. Immediately before and after the MRI experiments, subjects were asked to rate their subjective sensation of fatigue on the visual analogue scale (VAS) from 0 (no fatigue) to 10 (total exhaustion) [31].

### Magnetic resonance imaging

A time-course series of 364 (pre- and post-fatigue sessions), 1,804 (fatigue-inducing period for the CFS subjects), or 3,604 (fatigue-inducing period for the normal subjects) volumes were acquired using T2-weighted, gradient echo, echo planar imaging (EPI) sequences with a 3.0 tesla MR imager (Allegra; Siemens, Erlangen, Germany). Each volume consisted of 16 slices, each having a thickness of 6.0 mm, with a 1.2-mm gap between slices, to include the entire brain. The following parameters were used: time-interval, 1,000 ms; echo time, 30 ms; flip angle (FA), 60°; field of view (FOV), 19.2 cm; in-plane matrix size, 64 × 64 pixels; pixel dimensions, 3.0 × 3.0 mm. Magnetic shim was optimised so that a true in-plane resolution of 3.0 × 3.0 mm was realised. Tight but comfortable foam padding was placed around the subject's head to minimize head movement. For anatomical reference, T1-weighted fast-spin echo images (time-interval, 1,460 ms; echo time, 4.88 ms; FA, 8°; FOV, 19.2 cm; in-plane resolution, 0.9 × 0.8 mm; slice thickness, 3.6 mm; 16 axial slices covering the entire brain) were obtained from each subject with location variables identical to those of the EPI sequences. In addition, high-resolution whole-brain MRI images were obtained using a conventional T1-weighted, fast-spin echo sequence (time-interval, 1,970 ms; echo time, 4.38 ms; FA, 8°; FOV, 21.0 cm; in-plane resolution, 0.82 × 0.82 mm; slice thickness, 1.2 mm; 160 axial slices covering the entire brain).

### Data acquisition and statistical analyses

The first 4 volumes acquired in each MRI session were discarded due to unsteady magnetisation, and the remaining 360 (pre- and post-fatigue sessions), 1,800 (fatigue-inducing period for the CFS subjects), or 3,600 (fatigue-inducing period for the normal subjects) volumes were used for the analyses. Data were analysed using the Matlab [32-34] (Mathworks, Sherbon, MA) software package, implementing statistical parametric mapping (SPM99, Wellcome Department of Cognitive Neurology, London, UK). Following realignment, all images were coregistered to the high-resolution, 3-dimensional, T1-weighted MRI using the anatomical MRI with T1-weighted spin-echo sequences from identical locations. The parameters for affine and nonlinear transformation into a template of T1-weighted images (MNI template) were estimated from the high-resolution, 3-dimensional, T1-weighted MRI by using least squares means [34] already fit for a standard stereotaxic space [35]. These parameters were then applied to the coregistered fMRI data. The anatomically-normalised fMRI data were filtered using a Gaussian kernel of 8 mm (full-width at half-maximum) in the x, y, and z axes. We obtained responsiveness of auditory cortices by transient reduction of fMRI acquisition noise. The detailed method was described previously [29]. Briefly, we turned off slice readout gradient for 1 s periods; turning off the readout gradient reduced the frequency components from 107.2 to 88.8 dB, and the sound pressure was lowered to 1/10 and the sound intensity to 1/100 of the original levels, representing relatively silent periods during scans with an inter-trial interval of 13–17 s. The missing volumes were linearly interpolated with volumes immediately preceding and following the 'OFF' period. During these silent 'OFF' periods, we provided radiofrequency pulses in order to keep the magnetisation constant.

Statistical analyses were conducted at 2 levels. First, individual task-related activation was evaluated. Second, so that inferences could be made at a population level, individual data were summarised and incorporated into a random-effect model [36].

The signal was proportionally scaled by setting the whole-brain mean value to 100 arbitrary units in order to remove the global signal change. Expected signal changes caused by the tasks were modelled with a box-car function convolved with a hemodynamic response function and high-pass filtering [statistical parametric map (SPM) default calculated on the basis of trial frequencies]. Percent change in MR signal (percent signal change), relative to the global mean signal, was measured on a region-of-interest basis. The resulting set of voxel values for each comparison constituted a SPM of the t statistics (SPM{t}). The SPM{t} was transformed to the unit of normal distribution (SPM{Z}). The threshold for the SPM{Z} of individual analyses was set at P < 0.05 with a correction for multiple comparisons at the cluster level of the entire brain. [36]. The weighted sum of the parameters estimated in the individual analysis consisted of "contrast" images, which were used for the group analyses [36]. The contrast images obtained by individual analysis represented the normalised increment of the fMRI signal for each subject. SPM{t} and SPM{Z} for the contrast images were created as described above. Significant signal changes for each contrast were assessed by means of t statistics on a voxel-by-voxel basis [36]. The threshold for the SPM{Z} of group analyses was set at P < 0.05 with a correction for multiple comparisons at the cluster level of the entire brain [36]. The intensity threshold applied to the cluster-level statistics was set at P value less than 0.001, and the extent threshold in terms of number of voxels was more than 10 ones, respectively.

To evaluate attenuation of the brain activity responded to transient noise reduction during the fatigue session, we conducted regression analyses. The concept of this analytical method is to detect change of stimulus-locked (in this case, transient noise reduction) neural responsiveness in the entire brain. The detailed method was described previously [37]. Briefly, high and low pass filtered MR signals were realigned at the 'OFF' event, and then collected 15 scan points data from 1 scan point before the 'OFF' event. To normalise MR data (normalised peak activity) in each event, we calculated activity in each event divided by that in the first event. Then linear trend within each event was removed. To depict signal change across events, linear trend over time at each scan point was calculated with least square method. This evaluation was applied to all voxels, so that a contrast image containing slope estimate of every voxel was generated. The contrast image of 7th scan point was analysed in the present study because peak amplitude for hemodynamic response to transient noise reduction was shown [29]. One or two sample t-test was conducted for group analyses. In addition, 10-event moving-averaged MR signals of 7th scan point were plotted in the CFS and normal subjects, and compared the CFS patients with the normal subjects by covariance analyses.

## Results

**Figure 3:**
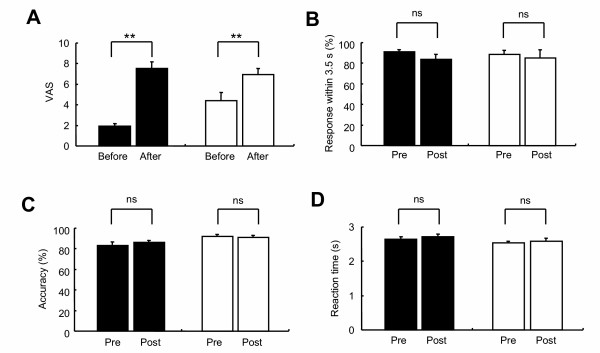
Subjective and behavioural results. (A) Results of subjective fatigue sensation. Just before (Before) and after (After) the fMRI experiments, subjects were asked to rate subjective sensation of fatigue on the visual analogue scale (VAS) from 0 to 10. (B-DS) Behavioural results. In the pre- (Pre) or post-fatigue (Post) sessions, subjects performed visual search or null trials for 6 min. During these sessions, percentage of the response within 3.5 sec (B), accuracy (C), and reaction time (D) were recorded. Closed columns, normal subjects; open columns, CFS subjects. **P < 0.01, significant difference; ns, no significant difference (paired t-test). Data are mean and SEM.

VAS scores for subjective sensation of fatigue were determined just before and after MRI experiments (Figure 3A). Before the experiments, the CFS patient group had significantly higher VAS scores than the normal group (CFS, 4.39 ± 2.02; normal, 1.94 ± 0.66; P = 0.011, unpaired t-test). Although two-way (2 × 2) repeated measures analysis of variance (ANOVA) of VAS score did not show significant main effect of group [F_(1,11) _= 5.54, P = 0.256], it revealed significant main effect of condition [F_(1,11) _= 106.30, P < 0.001] and condition × group interaction [F_(1,11) _= 14.98, P < 0.002]. Task performance was measured in the pre- and post-fatigue sessions (Figure 3B, C, D). In the pre-fatigue session, percentage of a response within 3,500 ms and accuracy in visual search trials were almost similar between the 2 groups (normal, 90.9 ± 5.0% and CFS, 88.6 ± 9.3%, P = 0.578; normal, 83.3 ± 9.3% and CFS, 92.2 ± 4.5%, P = 0.056, respectively; unpaired t-test). In the pre-fatigue session, reaction time in the visual search trials was also similar between the 2 groups (normal, 2.65 ± 0.18 s; CFS, 2.54 ± 0.12 s; P = 0.238, unpaired t-test). It therefore appeared that the effects of differences in task difficulty between the 2 groups were minimal. Repeated measures ANOVA of percentage of a response within 3,500 ms, accuracy, or reaction time in the visual search trials did not show main effect of condition, group, or condition × group interaction. Hence, task performance was not altered after the fatigue-inducing session in either the normal or CFS subjects.

**Figure 4:**
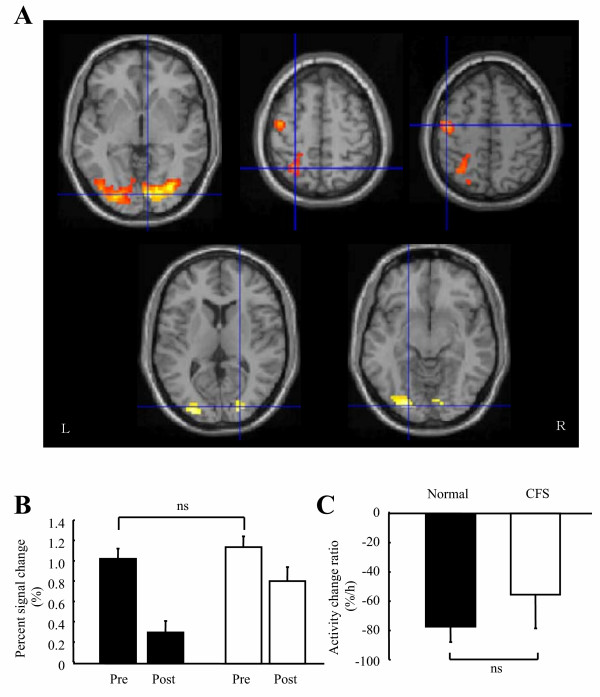
Activity related to visual search. (A) Statistical parametric maps activated by visual search (random-effect analysis of 13 subjects, P < 0.05, corrected). In order to identify brain regions activated by visual search, we calculated percent signal change evoked during present and absent trials as compared with that for null trial. Anatomical locations were mapped on axial template brains. Top: Sections passing through visual cortices (left), intraparietal sulcus (middle), and precentral gyrus (right) in the pre-fatigue session. Bottom: Sections passing through right visual cortex (left) and left visual cortex (right) in the post-fatigue session. Crossing line indicates the local activity maximum of each brain region. The right (R) and left (L) sides are indicated. (B) Activity in the visual cortices during the visual search in the pre- (Pre) and post-fatigue (Post) sessions of the normal and CFS patient groups. Magnitude of the activity was calculated as the peak signal change (%) averaged across the activated areas. (C) Activity change rate in the visual cortices during the visual search of the normal and CFS patient groups. Activity change rate (%/h) was calculated as change of activity in the visual cortices during the visual search from pre-fatigue to post-fatigue sessions divided by the session time (hour) of the fatigue-inducing period × 100. Data are mean and SEM. ns, no significant difference (unpaired t-test). Closed columns, normal subjects; open columns, CFS subjects.

In the pre-fatigue session, visual search was associated with the activation of bilateral visual cortices [Brodmann's areas (BA) 17, 18, 19], left superior and inferior parietal lobules (BA 7), and left precentral gyrus (BA 6) for all the subjects (Table 1 and Figure 4A). In the post-fatigue session, visual search was only associated with the activation of the visual cortices (BA 17, 18) (Table 1 and Figure 4A). Thus, in the pre- and post-fatigue session, we measured the activity of the visual cortices that had been activated during the visual search. Magnitude of the activity was calculated as the peak signal change (%) averaged across the activated areas. Activity in the visual cortices during the visual search was similar between the normal and CFS patient groups in the pre-fatigue session (1.02 ± 0.31% and 1.15 ± 0.26%, respectively; P = 0.428, unpaired t-test; Figure 4B). Since we could not obtain the time course of the activity of the visual cortices during the fatigue-inducing period, we evaluated the change rate of activity of these brain regions after the fatigue-inducing period. After the fatigue-inducing continual visual search task, the activity in the visual cortices was lower for the normal (pre-, 1.02 ± 0.31% and post-, 0.29 ± 0.30%, P < 0.001, paired t-test; Figure 4B) and that had a trend toward lower for the CFS subjects (pre-, 1.15 ± 0.28% and post-, 0.82 ± 0.34%, P = 0.078, paired t-test; Figure 4B), and the activity change rate, calculated as change of activity in the visual cortices during the visual search from pre-fatigue to post-fatigue sessions divided by the session time (hour) of the fatigue-inducing period × 100, in these brain regions was similar between the normal and CFS patient groups (normal, -76.4 ± 30.6%/h and CFS, -55.6 ± 56.8%/h, P = 0.418, unpaired t-test; Figure 4C).

**Figure 5:**
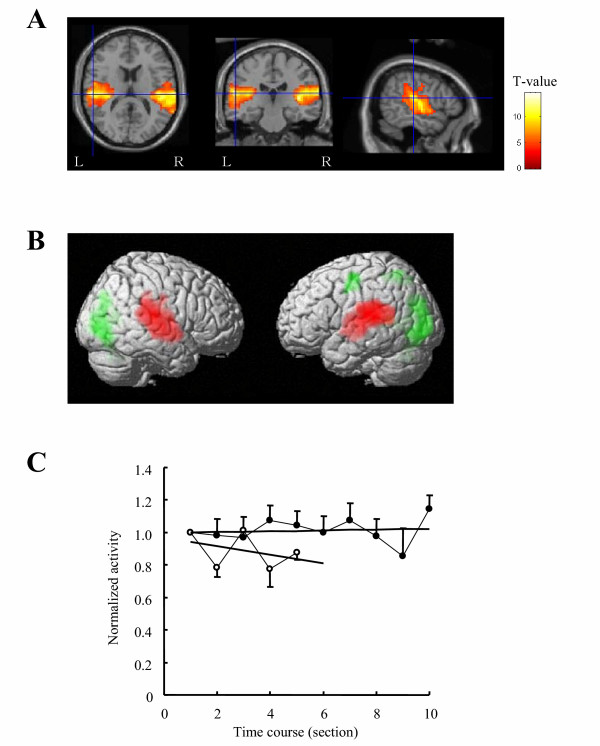
Activity related to transient reduction of fMRI acquisition noise. (A) Statistical parametric maps activated by noise reduction in the pre-fatigue session (random-effect analysis, P < 0.05, corrected). Anatomical locations were mapped on template brains. Axial (left), coronal (middle), and sagittal (left) sections passing through auditory cortices are shown. Crossing line indicates the local activity maximum of the brain regions. The right (R) and left (L) sides are indicated. (B) Statistical parametric maps activated by fMRI noise reduction (red) and visual search (green) in the pre-fatigue session (random effect analysis, P < 0.05, corrected). Combined statistical parametric maps were superimposed on surface-rendered high-resolution MRIs viewed from right and left. (C) Time course of normalised activity in the auditory cortices during fatigue-inducing period in the normal and CFS patient groups. To evaluate the time course of the auditory responses, we divided the fatigue-inducing period into 6-min sections, and measured activity of the brain regions that had been activated by transient reduction of fMRI noise in the pre-fatigue session. Magnitude of the activity was calculated as the peak signal change (%) averaged across the activated areas. Normalised activity was calculated as activity in a given section divided by that in the first section. Data are mean and SEM. Closed circles, normal subjects; open circles, CFS subjects.

In the pre-fatigue session, bilateral auditory cortices (BA 41, 42, and 22) were activated by transient reduction of fMRI acquisition noise for all the subjects (Table 2 and Figure 5A), which is consistent with the finding of a previous study [29]. In contrast, visual search was associated with the activation of the bilateral visual cortices (BA 17, 18, and 19), the left superior and inferior parietal lobules (BA 7), and left precentral gyrus (BA 6) in the pre-fatigue session (Table 1 and Figure 4A). The brain regions activated by this transient noise reduction did not overlap with those activated by the visual search (Figure 5B). This enabled us to record responses of task-independent brain regions. In addition, since fMRI acquisition noise was sporadically reduced independently of task trials, subjects could perform visual search task without or minimal perturbation for task trials.

We then investigated the time course of the responsiveness of the auditory cortices during the fatigue-inducing period. We divided the fatigue-inducing period (session time was 1 hour for the normal subjects and 30 min for the CFS subjects) into 6-min sections, and measured activity of the brain regions that had been activated by transient reduction of fMRI noise in the pre-fatigue session. Magnitude of the activity was calculated as the peak signal change (%) averaged across the activated areas. The activity of the auditory cortices in the pre-fatigue session was similar in the normal and CFS patient groups (0.253 ± 0.076% vs. 0.261 ± 0.060%, respectively; P = 0.840, unpaired t-test). We performed two-way repeated measures ANOVA of the activity, and evaluated group × time interaction. Since the normal group had 10 time points whereas CFS group had 5 time points, we evaluated only the first 5 time points. It revealed significant group × time interaction [F_(4,8) _= 3.46, P = 0.015]. In addition, the normalised activity change ratio [normalised activity was calculated as activity in a given section divided by that in the first section, and normalised activity change ratio was calculated from the slope of regression fitted to a scatter plot of the time course (section) against normalised activity] was lower in the CFS patient group than in the normal group (-0.026 ± 0.022 vs. 0.002 ± 0.018, P = 0.026, unpaired t-test; Figure 5C).

**Figure 6:**
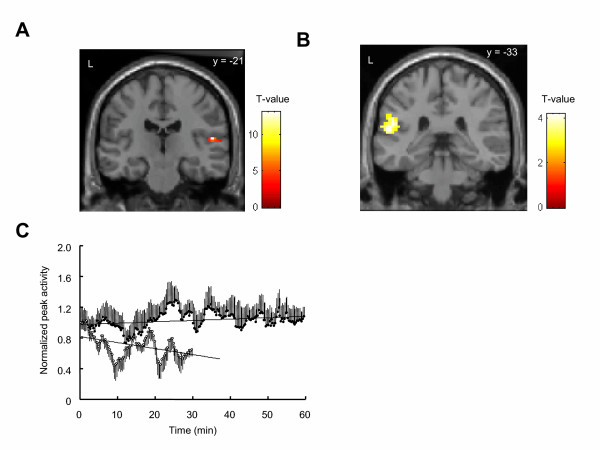
Activity related to noise reduction by regression analyses. Brain regions in which responsiveness to transient noise reduction was decreased in the CFS patients (A), and those in which responsiveness to transient noise reduction was decreased in the CFS patients relative to the normal subjects (B) during the fatigue session over 30 min (P < 0.05, uncorrected) are shown. Brain regions shown in Figure 6A and B are limited within auditory cortices. Anatomical locations were mapped on coronal template brains, with coordinate in mm from the anterior commissure. The left side (L) is indicated. (C) Time course of 10-event moving-averaged normalised peak activity of the left planum temporale. Normalised peak activity was calculated as shown in 'Materials and Methods' section. Linear regression lines are shown. Closed circles, normal subjects; open circles, CFS subjects. Data are mean and SEM.

In order to localise the brain region in which responsiveness to transient reduction of fMRI noise was prominently decreased in the CFS subjects relative to the normal subjects, we conducted regression analyses in the auditory cortices during the fatigue-inducing period over 30 min. Although there were no brain regions in which the responsiveness was decreased during the fatigue session in the normal subjects (Table 3), in the right planum temporale (BA 22), the responsiveness was decreased in the CFS patients (Table 3 and Figure 6A). A difference was observed in the left planum temporale between the 2 groups (BA 22; Table 3 and Figure 6B). Moving-averaged normalised activity confirmed this difference, and responsiveness to the transient noise reduction was significantly diminished in the CFS patients relative to the normal subjects during the fatigue session (Figure 6C).

**Figure 7:**
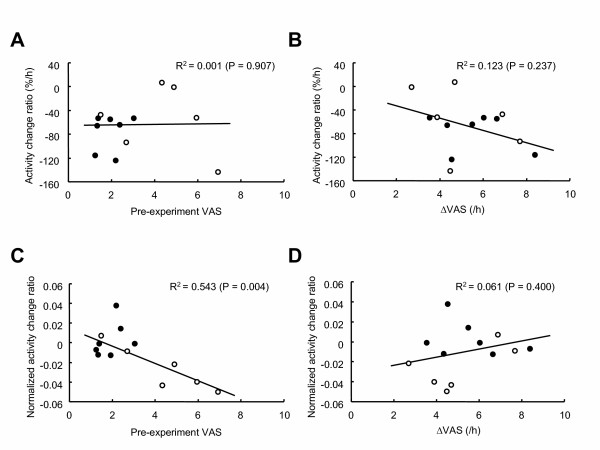
Subjective-activity correlations. Subjective scale correlations for the signal changes of the visual (A, B) and auditory (C, D) cortices are shown. Activity change rate or normalised activity change ratio were plotted against pre-experiment visual analogue scale score (pre-experiment VAS) (A, C) or change rate of visual analogue scale score after the experiment (ΔVAS) (B, D). Activity change rate was calculated as change of activity of the visual cortices during the visual search from pre-fatigue to post-fatigue sessions divided by the session time (hour) of the fatigue-inducing period. Normalised activity was calculated as activity of the auditory cortices in a given section divided by that in the first section. Normalised activity change ratio was calculated from the slope of regression fitted to a scatter plot of the time course (section) against normalised activity. Subjects were asked to rate subjective sensation of fatigue on the VAS from 0 to 10. Squared Pearson correlation (R^2^) and P value are displayed. Closed circles, normal subjects; open circles, CFS subjects.

Finally, we investigated the subjective correlations for the signal change of the visual and auditory cortices. Activity change rate in the visual cortices was neither correlated with the VAS score before the experiments nor change rate of VAS score per hour after the experiments for all the subjects (R^2 ^= 0.001, P = 0.907, Figure 7A; R^2 ^= 0.237, P = 0.123, Figure 7B, respectively). The normalised activity change ratio in the auditory cortices was negatively correlated with the VAS score before the experiments for all subjects (R^2 ^= 0.543, P = 0.004, Figure 7C). This correlation was stronger when limited in the CFS patient group (R^2 ^= 0.828, P = 0.012). The normalised activity change ratio in the auditory cortices was not correlated with the change rate of VAS score per hour after the experiments for all subjects (R^2 ^= 0.061, P = 0.400, Figure 7D).

## Discussion

In the present study, we demonstrated that the responsiveness in the task-dependent brain regions was decreased after the fatigue-inducing continual visual search task in the normal and CFS patient groups and that the decrement of the responsiveness in those brain regions was equivalent between the 2 groups. In addition, we found that, during the fatigue-inducing period, although responsiveness in the task-independent brain regions remained constant in the normal subjects, it was attenuated in the CFS patients. Moreover, rate of attenuation in the task-independent brain regions was positively correlated with the pre-experiment subjective sensation of fatigue as measured using a fatigue VAS.

After the fMRI experiments, the CFS and normal groups exhibited a significant increase in fatigue VAS score. This shows that both CFS and normal subjects had increased fatigue sensation by performing the continual visual search task, and that the task which we used in the fMRI experiments was valid as a fatigue-inducing task. We determined the number of targets for which subjects could provide correct answers to visual search trials in approximately 90% of cases. The CFS patients performed task trials with smaller number of targets relative to the normal subjects (19.0 ± 1.3 vs. 22.6 ± 2.0, P = 0.003, unpaired t-test). This suggests that the CFS subjects had difficulties for the task trials in terms of mental speed or motivation. In our study, although fatigue VAS score was increased, task performance was not altered after the fatigue-inducing task in the CFS and normal subjects. Therefore, task performance might not be a proper scale to measure the levels of CNS fatigue.

In the pre-fatigue session, visual search was associated with the activation of the visual cortices (BA 17, 18, and 19), superior and inferior parietal lobules (BA 7), and precentral gyrus (BA 6). As indicated in a review article on this subject [38], the activated brain regions in the pre-fatigue session of our study were almost similar to those reported previously by many researchers during many kinds of visual search trials using fMRI. We found decreased responsiveness of the visual cortices after the fatigue-inducing continual visual search task in the normal and CFS patient groups. However, the decrement of the activity in these brain regions was similar between the 2 groups. In addition, this decrement was not correlated with the fatigue VAS score before the experiments or change rate of fatigue VAS score after the experiments. These results suggest that reduction of the responsiveness of task-dependent brain regions is a common feature across normal and CFS subjects, and that decrement is caused not only by CNS fatigue but also by some confounds (e.g., mental efforts, attention, or learning).

In the pre-fatigue session, auditory cortices (BA 41, 42, and 22) were activated by transient reduction of the fMRI acquisition noise for 1 s, which is consistent with a previous finding [29]. The responsiveness to 1 s silent period mainly represents 'OFF'-set activity [29], and electrophysiological and fMRI 'OFF'-set responses imply increased activity in auditory cortical neurons following sound 'OFF'-set [39-43]. The brain regions activated by this transient noise reduction did not overlap with those activated by the visual search (Figure 5B). This enabled us to record responses of task-independent brain regions. In addition, since fMRI acquisition noise was sporadically reduced independently of task trials, subjects could perform visual search task without or with minimal perturbation for task trials.

The responsiveness in the auditory cortices was similar between the normal and CFS patient groups in the pre-fatigue session. However, during the fatigue-inducing period, although responsiveness of those brain regions was kept constant in the normal subjects, that was attenuated in the CFS patients. Hence, CFS may be characterised by the attenuation of the responsiveness to the stimuli not related to the fatigue-inducing task. Although the attenuation rate for the responsiveness of auditory cortices during the fatigue-inducing period was negatively correlated with the VAS score before the experiments, that was not correlated with the change rate of VAS score per hour after the experiments for all subjects. This suggests that attenuation rate for the responsiveness of task-independent brain regions is associated with the baseline level of fatigue sensation rather than change rate of fatigue sensation under the condition of fatigue.

Surprisingly, continual activation of task-dependent brain regions decreased the responsiveness of other brain regions not related to task trials in the CFS patients, and the mechanism remains to be clarified. Similar phenomenon can be seen during and after cortical spreading depression. Cortical spreading depression is a pronounced depolarisation of neurons and glia that spreads slowly across the cortex followed by a period of depressed electrophysiological activity [44]. A fMRI study demonstrated that signal amplitude, duration, and time to peak showed little recovery at 60 min post-induction of spreading depression, and analysis of spontaneous vasomotor activity suggested a breakdown of the neurovascular coupling relationship [45]. Of course, during the fatigue-inducing period, pronounced depolarisation of neurons and glia must not happen in the task-dependent brain regions, continual activation of some cortical regions may introduce breakdown of the neurovascular coupling relationship across other cortical regions in the CFS patients. Since CFS patients showed impaired vasomotor function [46], insult of CNS fatigue in the task-dependent brain regions may easily cause reduced responsiveness through breakdown of the neurovascular coupling relationship even in the task-independent brain regions. Alternatively, since change of responsiveness may be due to total changes of neurovascular coupling or neuronal activity [47-49], it is possible that reduced BOLD signal during fatigue-load indicates reduced activation of auditory cortical neurons. In any case, CNS fatigue may attenuate not only neurovascular coupling relationship and/or neuronal activities of the task-dependent brain regions but also those of other brain regions through interneuronal and intercortical connections.

During the fatigue-inducing period, responsiveness in the task-independent brain regions was reduced with slow fluctuations in the CFS patients. Low frequency (0.1 Hz) fluctuations of signal intensity have been shown in fMRI studies [50,51], and the fluctuations are likely to be vasomotor in origin [51]. Thus, CNS fatigue may induce breakdown of feedback in neurovascular coupling followed by an increase in the magnitude and a reduction of fluctuations in the CFS patients.

In the right planum temporale, the responsiveness was decreased in the CFS patients, and in the left planum temporale, reduction of responsiveness by transient reduction of fMRI noise was shown in the CFS patients relative to the normal subjects during the fatigue-inducing period over 30 min. It seems that the reduced level of the responsiveness was literalised among task-independent brain regions. However, although not statistically significant, those phenomena could be seen in the opposite sides (data not shown). We assume that, although reduced level of the responsiveness is heterogeneous among task-independent brain regions, reduction can be seen among all the task-independent brain regions. Further studies would answer this problem.

Our findings have valuable clinical implications for CFS. CFS is currently diagnosed on a symptomatic basis rather than from physical examinations or routine laboratory findings [1]; no physiological diagnostic tests have yet been established. Since we could to some extent distinguish CFS patients from normal subjects by comparing the attenuation rate for the responsiveness of task-independent brain regions during the fatigue-inducing period using fMRI, we believe that this new method could facilitate the diagnosis of CFS. In addition, since attenuation rate was positively correlated with pre-experiment VAS scores in the CFS patients (R^2 ^= 0.828, P = 0.012), we propose this parameter as a new objective and quantitative scale to measure the severity of CFS.

This study had 3 limitations. First, the auditory cortices were the only task-independent brain regions evaluated. Further studies are needed to determine the responsiveness of other brain regions. Second, as a sample size was small in this study, larger numbers of subjects need to be investigated before our findings can be generalised. Finally, as for the fatigue session, there existed time differences between the CFS and normal subjects. After considering the physical and mental condition of the CFS patients, we determined that 30 min was a suitable period for these patients to perform the trials. In contrast, in order to have similar level of fatigue sensation relative to the CFS subjects after the MRI experiments, it was essential for the normal subjects to perform task trials for 1 hour. However, results of regression analyses during the fatigue-inducing period over 30 min ensure the validity of the reduced responsiveness of the task-independent brain region in the CFS patients.

## Conclusion

In conclusion, CFS may be characterised by the attenuation of brain responsiveness not directly related to task trials. Our findings provide a new perspective on the pathophysiology of CFS and the neural basis of CNS fatigue.

## List of abbreviations

ANOVA, analysis of variance; BA, Brodmann's area; CFS, chronic fatigue syndrome; CNS, central nervous system; EPI, echo planar imaging; FA, flip angle; fMRI; functional magnetic resonance imaging; FOV, field of view; MRI, magnetic resonance imaging; VAS, visual analogue scale

## Competing interests

The author(s) declare that they have no competing interests.

## Authors' contributions

MT carried out the fMRI studies, participated in the study design, analysed the fMRI data, and drafted the manuscript. NS participated in the study design, performed the fMRI studies, and helped to draft the manuscript. TO performed the fMRI studies and analyzed the fMRI data. KM performed the fMRI studies and analysed the fMRI data. TS performed the fMRI studies. HT analysed the fMRI data and helped to draft the manuscript. DS performed the fMRI studies. HO participated in the study design, analysed the fMRI data, and helped to draft the manuscript. HK was responsible for medical assessment of the patients and assisted the execution of fMRI studies. YW participated in the study design and helped to draft the manuscript. All authors read and approved the final manuscript.

## Pre-publication history

The pre-publication history for this paper can be accessed here:



## Figures and Tables

**Table 1:** Brain regions activated by visual search in the pre- and post-fatigue sessions.

Location	Side	Brodmann's area	Cluster size (voxels)	Coordinates (mm)	Z- value
**Pre-fatigue session**					
Occipital cortices	R/L	17, 18, 19	1799	9 -84 -3	5.79
Precentral gyrus	L	6	103	-45 -9 54	4.77
Superior parietal lobule/inferior parietal lobule	L	7	79	-27 -57 57	4.07
**Post-fatigue session**					
Occipital cortex	R	17, 18	246	-21 -87 -9	4.18
Occipital cortex	L	17, 18	77	24 -87 9	4.32

Random-effect analysis of 13 subjects, P < 0.05, corrected for the entire search volumes.

x, y, z: Stereotaxic coordinates of peak of activated clusters.

**Table 2:** Brain regions activated by transient reduction of fMRI acquisition noise in the pre-fatigue session.

Location	Side	Brodmann's area	Cluster size (voxels)	Coordinates (mm)	Z- value
Transverse temporal gyri/superior temporal gyri	R	41, 42, 22	1407	57 -24 15	5.63
Transverse temporal gyri/superior temporal gyri	L	41, 42, 22	1280	-39 -21 6	5.85

Random-effect analysis of 13 subjects, P < 0.05, corrected for the entire search volumes.

x, y, z: Stereotaxic coordinates of peak of activated clusters.

**Table 3:** Brain regions in which responsiveness to transient noise reduction was decreased during 30-min fatigue session.

Location	Side	Brodmann's area	Cluster size (voxels)	Coordinates (mm)	Z- value
**Normal**					
Not detectable					
**CFS**					
Transverse temporal gyri*	R	22	22	54 -21 12	4.08
**CFS > Normal**					
Transverse temporal gyri*	L	22	65	-54 -33 18	3.18

Limited within auditory cortices.

*P < 0.05, uncorrected for the entire search volumes.

x, y, z: Stereotaxic coordinates of peak of activated clusters.
